# Quantitative Analysis of the Effect of Neuromuscular Blockade on Motor-Evoked Potentials in Patients Undergoing Brain Tumor Removal Surgery: A Prospective, Single-Arm, Open-Label Observational Study

**DOI:** 10.3390/jcm13154281

**Published:** 2024-07-23

**Authors:** Dongwoo Chae, Hyun-Chang Kim, Hun Ho Park, Jihwan Yoo, Yoon Ghil Park, Kyu Wan Kwak, Dawoon Kim, Jinyoung Park, Dong Woo Han

**Affiliations:** 1 Department of Pharmacology, Yonsei University College of Medicine, 50 Yonsei-ro, Seodaemun-gu, Seoul 03722, Republic of Korea; dongy@yuhs.ac; 2 Department of Anesthesiology and Pain Medicine and Anesthesia and Pain Research Institute, Yonsei University College of Medicine, 50 Yonsei-ro, Seodaemun-gu, Seoul 03722, Republic of Korea; onidori@yuhs.ac; 3 Department of Neurosurgery, Brain Tumor Center, Gangnam Severance Hospital, Yonsei University College of Medicine, 211 Eonju-ro, Gangnam-gu, Seoul 06273, Republic of Korea; nshhp@yuhs.ac (H.H.P.); jhy8486@yuhs.ac (J.Y.); 4 Department of Rehabilitation Medicine, Gangnam Severance Hospital, Yonsei University College of Medicine, 211 Eonju-ro, Gangnam-gu, Seoul 06273, Republic of Korea; drtlc@yuhs.ac (Y.G.P.); kyuwankw@yuhs.ac (K.W.K.); s2loos@yuhs.ac (D.K.); mdjyp@yuhs.ac (J.P.)

**Keywords:** brain neoplasm, electric impedance, motor-evoked potential, neuromuscular blockers, transcranial magnetic stimulation

## Abstract

**Background**: We aimed to elucidate the quantitative relationship between the neuromuscular blockade depth and intraoperative motor-evoked potential amplitudes. **Methods***:* This prospective, single-arm, open-label, observational study was conducted at a single university hospital in Seoul, Korea, and included 100 adult patients aged ≥19 years undergoing brain tumor removal surgery under general anesthesia. We measured the neuromuscular blockade degree and motor-evoked potential amplitude in the deltoid, abductor pollicis brevis, tibialis anterior, and abductor hallucis muscles until dural opening. **Results**: The pharmacokinetic-pharmacodynamic model revealed the exposure-response relationship between the rocuronium effect-site concentration and motor-evoked potential amplitudes. The mean motor-evoked potential amplitudes decreased proportionally with increasing neuromuscular blockade depth. As the mean amplitude increased, the coefficient of variation decreased bi-exponentially. The critical ratio of the first evoked response to the train-of-four stimulation (T1)/control response (Tc) thresholds beyond which the coefficient of variation exhibited minimal change were found to be 0.63, 0.65, 0.68, and 0.63 for the deltoid, abductor pollicis brevis, tibialis anterior, and abductor hallucis muscles, respectively. **Conclusions**: Our results reveal that the motor-evoked potential amplitude exhibits deterioration proportional to the degree of neuromuscular blockade. In light of the observed bi-exponential decline of the coefficient of variation with the motor-evoked potential amplitude, we recommend maintaining a T1/Tc ratio higher than 0.6 for partial neuromuscular blockade.

## 1. Introduction

Motor-evoked potentials (MEPs) can be monitored to detect neurological injuries that occur during surgeries. The most significant MEP parameter is its amplitude, which quantifies the signal strength. The warning criterion for intraoperative MEP monitoring is an amplitude decrease of >50% from the baseline [1].

MEPs are sensitive to anesthetics. A neuromuscular blockade (NMB) can cause the reduction or complete loss of MEPs [2]. The optimal NMB for monitoring MEPs is controversial. The use of NMBs should be avoided during MEP monitoring, as it can affect the results. Regarding MEP amplitude and variability, no NMB was more desirable than any level of partial NMB [2]. Without an NMB, however, higher anesthetic dosages are required to restrict patient movement, resulting in an increased demand for vasopressors. An NMB is beneficial in facilitating muscle retraction and preventing patient movement. Some investigations suggest that partial paralysis may be acceptable [3,4]. Due to these advantages, some surgeons, neurophysiologists, and anesthesiologists prefer using a partial NMB [4,5]. Therefore, predicting the correlation between the degree of NMB and MEP signals is necessary, enabling their adequate interpretation. Although previous studies have attempted to determine the effects of partial muscle relaxation via an NMB on MEP amplitudes, the results remain controversial [2,3,4,5].

To determine the necessary extent of partial relaxation, establishing a quantitative dose-response relationship between the muscle relaxation degree via NMB and MEP amplitude changes is essential.

We aimed to outline the correlation between the depth of NMB and MEP amplitude, considering both the typical value and within-subject variability (WSV). The ratio of the first evoked response (T1) to train-of-four (TOF) stimulation to the maximum electromyographic amplitude of the twitch height before rocuronium infusion, considered the control response (Tc), that is the T1/Tc, was used to indicate NMB depth. The ultimate aim was to identify the critical T1/Tc level to ensure the reliable interpretation of MEPs.

## 2. Materials and Methods

### 2.1. Participants and Data Collection

This prospective, single-arm, open-label observational study enrolled 100 adults. The clinical trial review board of the Yonsei University Gangnam Severance hospital in Seoul, Korea, approved this trial (Document no. 3-2020-0511; December 2020). This study was registered at ClinicalTrials.gov (NCT04768400; February 2021).

Written informed consent was acquired before patient enrolment. The inclusion criteria entailed patients (1) aged ≥ 19 years undergoing brain surgery under general anesthesia and had provided informed consent; (2) with American Society of Anesthesiologists’ Physical Status classes I–III; and (3) scheduled for MEP monitoring. The exclusion criteria were as follows: (1) central or peripheral neuromuscular disorders such as cerebral palsy, myasthenia gravis, acute spinal cord injury, and neurological shock; (2) pre-existing sensory or motor neurological deficits; (3) allergies to propofol, remifentanil, or rocuronium; and (4) the presence of cardiac pacemakers.

After pre-oxygenation with 100% O_2_, propofol (4 µg/mL) and remifentanil (4 ng/mL) were infused using a target-controlled infusion pump (Orchestra, Fresenius Kabi, Bad Homburg, Germany) for anesthetic induction. Propofol was administered using the Schneider model, targeting the effect site concentration. Similarly, remifentanil was infused using the Minto model, also targeting the effect site concentration. The infusion pump was calibrated and programmed according to the patient’s weight, height, age, and gender to ensure the accurate delivery of the anesthetic agents. All the settings were double-checked to ensure their accuracy and safety before the patient’s arrival in the operating room. Before the infusion of the neuromuscular blockers, the baseline twitch response was recorded using a neuromuscular transmission module (TwitchView Electromyography; Blink Device Company, Seattle, WA, USA). The optimal stimulus current for obtaining the maximum response of the abductor pollicis muscle was recorded. The maximum electromyographic amplitude of the twitch height of the first evoked response to TOF stimulation (T1) before rocuronium infusion was considered the control response (Tc). The T1/Tc ratios of the participants were monitored every 30 s. Rocuronium 0.6 mg/kg was administered to perform tracheal intubation. The effect-site concentrations of propofol (3–5 µg/mL) and remifentanil (3–8 ng/mL), administered for anesthetic maintenance, were controlled at the discretion of the attending anesthesiologists. Body temperature was monitored using an esophageal temperature probe. Hypotension was defined as a decrease in the mean arterial pressure of >20% of the preoperative value and was treated with an infusion of phenylephrine and ephedrine. If the heart rate decreased to <50 beats per minute, atropine 5 mg was infused. All the patients were administered normal saline (2 mL/kg/h), and blood loss and urine output were replaced using a balanced crystalloid solution (Plasma Solution A, HK inno.N Corporation, Seoul, Republic of Korea).

### 2.2. Outcome Measures

#### Intraoperative MEP Monitoring

Intraoperative MEP monitoring was performed by a skilled technician under the supervision of an experienced physiatrist using an intraoperative monitoring system (Neuromaster MEE-1000; Nihon Kohden, Tokyo, Japan). The MEPs were evoked by transcranial electrical stimulation consisting of a six-stimulus train (square wave; pulse duration, 0.05 ms; interstimulus interval, 3.0 ms). Every stimulus had a uniform intensity of 300 Mv. The interhemispheric montages of C3/C4 and C4/C3 were used to evoke MEPs. Subdermal recording electrode pairs were inserted in the deltoid, abductor pollicis brevis, tibialis anterior, and abductor hallucis muscles bilaterally. The MEPs were periodically evoked until dural opening, and peak-to-peak amplitudes were recorded at each 10% T1/Tc increment and each recording point. The missing data regarding the amplitude of MEPs will be replaced using regression imputation. The propofol and remifentanil target concentrations, body temperature, mean blood pressure, heart rate, and cardiac index were recorded.

### 2.3. Statistical Analysis

#### 2.3.1. Pharmacokinetic–Pharmacodynamic Modelling of Rocuronium

To characterize the exposure–response relationship of rocuronium with respect to both the T1/Tc and MEP amplitude, we utilized a population pharmacokinetic–pharmacodynamic (PKPD) modelling approach. We applied a PKPD model of rocuronium previously published by Kleijn et al. to predict the rocuronium concentration after dosing [6]. The pharmacokinetic parameters employed for the modelling are listed in Table S1. The model equations are provided in the Supplementary Material.

#### 2.3.2. Assessment of Within-Subject Variability (WSV) of MEP Amplitudes

WSV in MEP amplitudes was estimated from the actual MEP amplitude data for the four monitored muscles. The mean (μ) and standard deviation (σ) of the MEP amplitudes were calculated, and the coefficient of variation (CV) was determined as σ/μ. We further examined the functional relationship between μ and σ using a nonlinear mixed-effects modelling framework.

#### 2.3.3. Software

Monolix version 2023R1 (https://lixoft.com/products/monolix/), accessed on 1 June 2023, was used for developing the population PKPD model of T1/Tc and MEP amplitude. R version 4.3.1 and RStudio were used for data wrangling, exploratory data analysis, preparation of the modeling dataset, post-hoc analysis based on the PKPD model estimates, and visualization. The following R libraries were employed, among others: tidyverse, ggplot2, reshape2, plyr, and VIM.

## 3. Results

Of the 102 patients screened from March 2021 to May 2022, 100 participated in the study, after excluding two patients who did not agree to participate (Figure S1). The characteristics of the study participants, including demographic information, concomitant diseases, and anesthetic characteristics are shown in Table 1.

### 3.1. Exploratory Data Analysis

The MEP amplitudes were measured repeatedly for four muscles: deltoid, abductor pollicis brevis, tibialis anterior, and abductor hallucis muscles, as the T1/Tc gradually returned to baseline levels. The mean (μ) and standard deviation (σ) of MEP amplitudes were calculated and denoted using subscripts for specific muscles (e.g., $\mu_{abductor\;pollicis\;brevis}$ and $\sigma_{abductor\;pollicis\;brevis}$).

**Figure 1:**
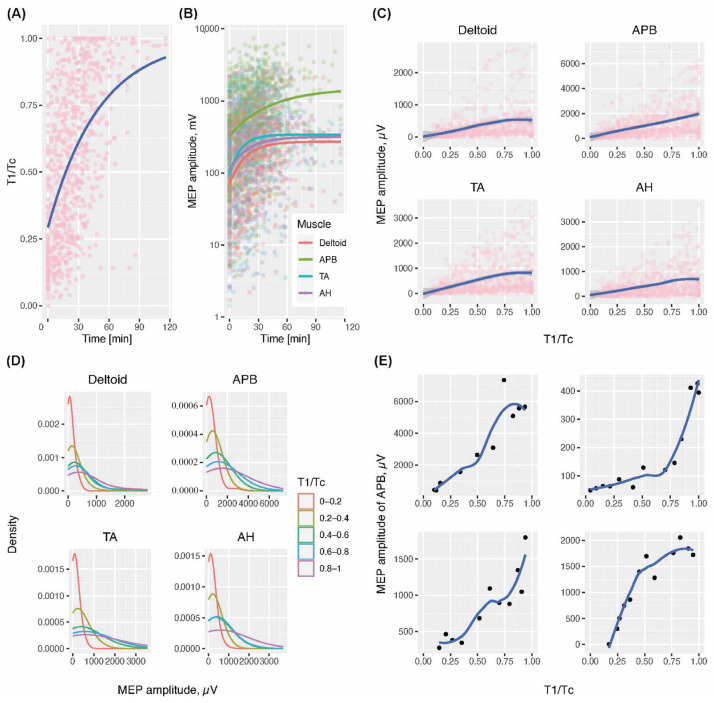
(**A**) The recovery trajectory of the T1/Tc and μ. (**B**) The recovery trajectories of the T1/Tc and μ of the four muscles. (**C**) The quantitative relationship between the μ and T1/Tc. (**D**) Distributional characteristics of μ for the four muscles conditioned on different T1/Tc intervals. (**E**) Individual-level relationships between μ and T1/Tc in four different individuals. T1, first evoked response to train-of-four stimulation; Tc, control response before rocuronium infusion; MEP, motor-evoked potential; Del, deltoid; APB, abductor pollicis brevis; TA, tibialis anterior; AH, abductor hallucis; µ, mean MEP amplitude.

Both the T1/Tc and μ were the most suppressed at the beginning of MEP monitoring, which progressively returned to baseline levels. According to the known duration of rocuronium action of 30–90 min, the T1/Tc and μ recovered completely within two hours for most patients (Figure 1A,B) [7]. The μ value differed considerably across the monitored muscles, with the average decrease in the order of the abductor pollicis brevis, tibialis anterior, abductor hallucis, and deltoid muscles (Figure 1B).

For all muscles, a positive correlation was observed between the μ and T1/Tc (Figure 1C). The mean amplitudes were consistently higher than the median amplitudes, with a 1000-fold difference between the maximum and minimum (Table S2). Additionally, the μ demonstrated significant between-subject variability (BSV) and a right-skewed distribution (Figure 1D).

On an individual level, the μ-T1/Tc curves varied substantially. Figure 1E illustrates the $\mu_{APB}$-T1/Tc curves pertaining to a random sample of four individuals in whom at least 10 repetitive measurements and a T1/Tc range exceeding 0.75 were obtained.

### 3.2. Prediction of T1/Tc and MEP Amplitudes through PKPD Modelling

**Figure 2:**
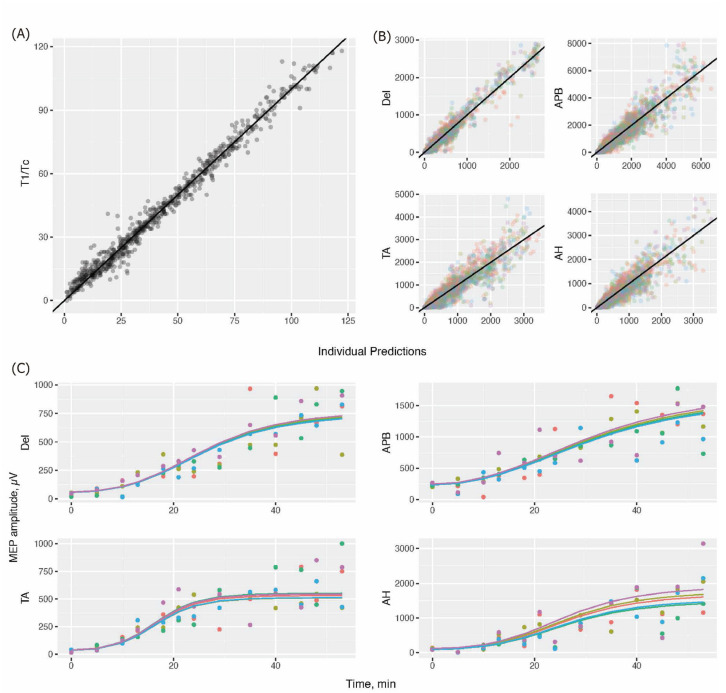
Observed and predicted values of the T1/Tc (**A**) and MEP amplitude (**B**). Measurements acquired from different sensors were distinguished using different colors. (**C**) Observed (dot) and predicted (line) MEPs overlaid in a randomly selected patient, with different colors representing measurements from different recording electrodes. T1, first evoked response to train-of-four stimulation; Tc, control response before rocuronium infusion; MEP, motor-evoked potential; Del, deltoid; APB, abductor pollicis brevis; TA, tibialis anterior; AH, abductor hallucis.

A PKPD model was developed for rocuronium, addressing both T1/Tc and MEP amplitudes (μ). The effect-site concentration of rocuronium, $C_{e}$, was correlated with the T1/Tc and μ through an inhibitory sigmoid $E_{max}$ model (see Supplementary Material). The estimation results are presented in Table 2. Strong agreement was observed between individual predictions and observations across all endpoints (Figure 2A,B). When the observed and predicted MEPs pertaining to a randomly chosen individual were overlaid on a single plot, our model suitably depicted MEP recovery over time (Figure 2C).

Substantial BSV was observed in the baseline μ, ranging from 80–110%. No significant covariates that could account for the baseline BSV were identified. The WSV of the baseline μ displayed the greatest magnitude in the abductor hallucis muscle (0.124), followed by the tibialis anterior (0.0915), abductor pollicis brevis (0.0549), and deltoid (0.0436) muscles (Table 2). Consistent with the preliminary results, the highest baseline μ was observed in the abductor pollicis brevis, followed by the tibialis anterior, abductor hallucis, and deltoid muscles, although the difference between the tibialis anterior and abductor hallucis muscles was minimal. Notable BSV was found for the half-maximal effective concentration (EC_50_), indicating that the rocuronium concentration linked with half-maximal suppression of the μ can differ considerably between patients. Conversely, the BSV of the EC_50_ associated with the T1/Tc ratio was significantly smaller.

In a typical individual, the abductor hallucis muscle is the most sensitive muscle to rocuronium, as indicated by the smallest EC_50_, followed by the tibialis anterior, abductor pollicis brevis, and deltoid muscles. However, the BSV related to the EC_50_ was greatest for the abductor hallucis muscle. When averaged across all muscles, the EC_50_ and Hill coefficients (2.495 and 2.18, respectively) were similar to those associated with the T1/Tc, suggesting that the μ could demonstrate an overall linear dependence on the T1/Tc.

### 3.3. Model Simulation Provides Potential Explanation for Diverse Shapes of μ-T1/Tc Curves

Monte Carlo simulations in 100 virtual subjects successfully replicated the variable shapes of μ-T1/Tc curves in diverse individuals (Figure S2). A mathematical analysis of Equation (S3) (see Supplementary Material) revealed that the relative magnitudes of the EC_50_ and Hill coefficient between the μ and T1/Tc (Figure S3) are pivotal factors for determining the shapes of μ-T1/Tc curves. Given the same Hill coefficient, a lower (higher) EC_50_ of the μ compared to the T1/Tc predicts a μ-T1/Tc curve that is convex (concave) upwards. When the EC_50_ values are the same, a higher Hill coefficient of the μ in relation to the T1/Tc predicts an increasingly sigmoid shape of a μ-T1/Tc curve, with the maximum slope at the T1/Tc of 0.5. A relatively lower Hill coefficient would also lead to increased sigmoidicity; however, in a pattern that mirrors the former with respect to the line of unity, the slope is minimal at a T1/Tc of 0.5.

All five qualitatively distinct shapes (linear, convex upwards, concave upwards, and the two sigmoid types) appeared in our data, corroborating the plausibility of our model in explaining the different patterns observed in the μ-T1/Tc curves.

### 3.4. CV Decreases Bi-Exponentially with Mean MEP Amplitude

**Figure 3:**
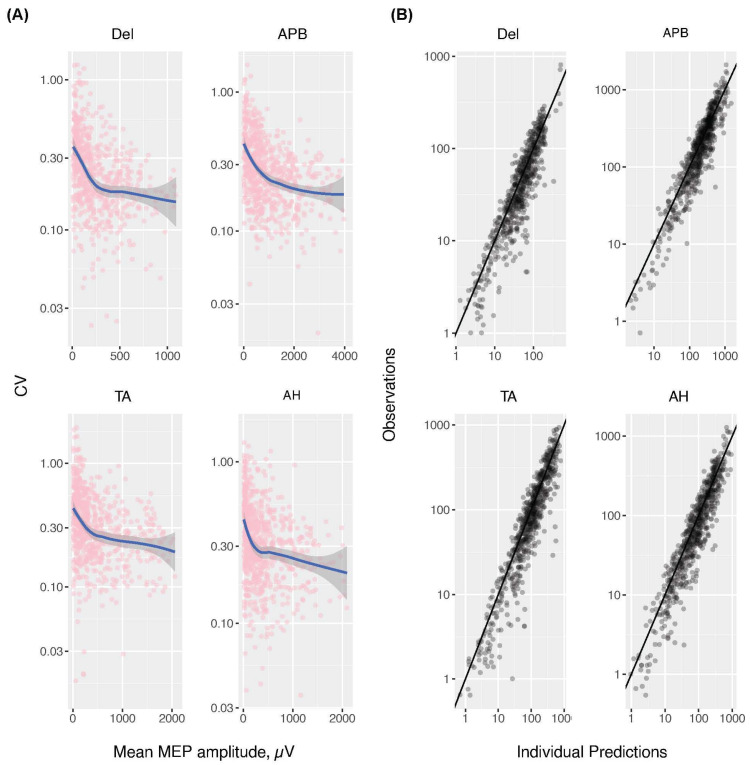
(**A**) The CV of the MEP amplitude is inversely correlated with the mean MEP amplitude. (**B**) Observed versus predicted standard deviations in the bi-exponential model. CV, coefficient of variance; Del, deltoid; APB, abductor pollicis brevis; TA, tibialis anterior; AH, abductor hallucis; MEP, motor-evoked potential.

The graphical exploration of the log-transformed CV versus the μ suggested a bi-exponential decline in the CV with the μ. The rapid decline phase concluded at MEP amplitudes of approximately 250, 1000, 500, and 250 μV in the deltoid, abductor pollicis brevis, tibialis anterior, and abductor hallucis muscles, respectively (Figure 3).

To quantitatively define the relationship between the CV and μ, we conducted a nonlinear mixed effects regression of the σ on the μ by fitting the mono-exponential (Model I) and bi-exponential models (equations are presented in the Supplementary Material). The parameters estimated were the maximum amplitudes of the two exponentials ($\alpha_{1}$, $\alpha_{2}$) and the MEPs associated with their 50% reduction ($\mu_{50,1}$, $\mu_{50,2}$). The theoretical CV at a mean MEP amplitude of zero is the sum of the maximum amplitudes of the exponential functions (i.e., $\alpha_{1}$ + $\alpha_{2}$). Smaller values of $\mu_{50,1}$ and $\mu_{50,2}$ predict a faster reduction of CV at higher mean MEP amplitudes.

A comparison of the Akaike information criteria between the two models revealed that for all four muscles, the bi-exponential model yielded a significantly better fit than the mono-exponential model. The estimation results of the bi-exponential model are presented in Table 3. The asymptotic maximum CV, expected under the μ approaching zero with complete NMB, was highest in the abductor hallucis muscle (55%), followed by the tibialis anterior (51%), abductor pollicis brevis (48%), and deltoid (41%) muscles, consistent with the order of WSV of the μ estimated in the PKPD model (Table 2).

The T1/Tc yielding critical MEP amplitudes of 250, 1000, 500, and 250 μV in the deltoid, abductor pollicis brevis, tibialis anterior, and abductor hallucis muscles were 0.63, 0.65, 0.68, and 0.63, respectively, calculated using Equation (S6) (see Supplementary Material) based on the estimated parameters. Alternatively, we calculated the MEP amplitudes corresponding to different target CVs by solving Equation (S9) (Supplementary Material).

Table 3 presents the results for the target CVs of 25%, 30%, and 35%. Given the commonly used intraoperative warning criteria of a 50% decline in MEP amplitude, targeting a CV of 25% was a plausible objective as ±2 standard deviations would yield an error margin of ±50%. In the deltoid and abductor pollicis brevis muscles, the T1/Tc values associated with a CV of 25% were 0.66 and 0.63, respectively. However, for the tibialis anterior and abductor hallucis muscles, the target CV of 25% was not attainable. Setting a target CV at 30%, considering the relatively higher CV in these muscles, resulted in T1/Tc thresholds of 0.66 and 0.67, respectively.

## 4. Discussion

Our research validated the inhibitory effect of NMB on MEP amplitudes. Contrary to previous findings [2,3,8], we could not identify a clear T1/Tc threshold linked to achieving a plateau in the MEP amplitude. In general, we observed an approximately linear dependence of the MEP amplitude on the T1/Tc. These findings suggest that MEP amplitudes are less reliable with even a slight residual effect of NMB, compared with those without any NMB. Moreover, the T1/Tc should be considered when interpreting the MEP change when MEPs are recorded during fluctuating NMB levels.

Previous studies have suggested that MEP amplitudes persist even under partial NMB [2,3,8]. In previous animal studies of transcranial magnetic MEP analysis in 10 female monkeys, the relationship between the μ and T1/Tc showed a positive correlation and achieved a plateau as the T1 approached 1.0, regardless of atracurium or vecuronium administration [3,8]. In our study with a larger sample size, the μ increase did not plateau at a critical T1/Tc level. The μ increase, however, demonstrated an approximately linear dependence on the T1/Tc. Sloan et al. proposed that NMB exerts a more substantial effect on peripheral versus central stimulation [2,4,5,8]. A recent study suggested a target T1/Tc of 0.5 for partial NMB based on negligible differences in MEP amplitude relative to no NMB [2]. The MEP amplitude, however, was largest in the group with no NMB compared with any level of partial NMB investigated and the authors recommended no muscle relaxation over partial NMB [2]. Our finding is in line with this recent previous investigation [2], confirming a linear relationship between the T1/Tc and MEP amplitudes in our investigation.

Instead of relying on an empirical regression analysis relationship between the MEP amplitude on the T1/Tc, we used prior information on rocuronium PKPD to elucidate the relationship between rocuronium exposure and MEP amplitudes [6]. This approach provided better mechanistic insight, successfully recreating the nonlinear dependence of MEP amplitudes on the T1/Tc observed in several patients.

The relationship between the T1/Tc and MEP amplitude varied among individuals and ranged from linear to concave, convex, and sigmoid shapes. We found notable differences in the reference MEP amplitudes among different muscle groups, with the abductor pollicis brevis and deltoid muscles showing the highest and lowest mean amplitudes, respectively. Intermuscular and individual differences should be considered when interpreting MEP amplitudes. In the inhibitory relationship with the MEP amplitude for rocuronium *C_e_* for each muscle (abductor hallucis, tibialis anterior, abductor pollicis brevis and deltoid muscles), the population mean EC_50_ values for rocuronium were 1.83, 2.46, 2.64, and 3.05 µM, respectively. The lower extremity muscles (abductor hallucis and tibialis anterior muscles) were more sensitively inhibited by rocuronium than the upper extremity muscles (abductor pollicis brevis and deltoid muscles), which is consistent with previous results [2]. This finding suggests that the muscle relaxant dose used should be reduced, especially for MEP monitoring in the lower extremities. These intermuscular differences in the MEP amplitude may be multifactorial and include motor unit count, muscle fibers per motor unit, sarcomere length, muscle tension in the surgical position, muscle size, and architecture.

Even after stratifying for different muscle locations, the random between-subject variability (BSV) of MEP amplitudes remained substantial, making it challenging to predict MEP amplitudes for individual patients a priori. This suggests that MEP amplitudes should be interpreted relative to the individual’s baseline levels. Fortunately, the within-subject variability (WSV) is substantially lower than BSV, although it depends sensitively on the MEP amplitude. The coefficient of variation (CV), defined as WSV (measured in standard deviations) divided by the mean MEP amplitude, was found to decrease at higher MEP amplitudes. Therefore, accurate MEP monitoring requires understanding the critical relationship between the noise level (CV) and the observed MEP amplitude.

For reliable MEP monitoring, the amplitude should be higher, and the CV should be smaller. An increased CV can increase the possibility of false-positive or false-negative ratios, which lowers the reliability of the MEP data. A significant discovery of our study is the evident bi-exponential decline in the CV with mean MEP amplitudes. This result corroborates the NMB effect on the MEP CV values reported in previous studies but distinguishes itself by developing a predictive model using nonlinear mixed-effects regression [2]. This pattern implies that partial NMB yielding MEP amplitudes higher than a critical threshold would result in small CV increases. By understanding the relationship between the T1/Tc and MEP amplitude, we determined the T1/Tc cut-off necessary for reliable MEP interpretation. Despite substantial differences in absolute MEP amplitudes, the critical T1/Tc level permitting reliable MEP interpretation was 0.6–0.7 in all four muscles. Hence, we recommend a T1/Tc higher than 0.6 when NMB is necessary intraoperatively. Based on a sufficient number of prospective data and analysis with population PKPD modelling, this study could provide more detailed guidelines than recent retrospective research, which concluded that a TOF ratio of 0.75 or higher is necessary for feasible MEP detection through subgroup analysis with segmented TOF ratio intervals [9]. Our findings show that earlier research on the acquisition of the MEP waveform, which is still used by some institutions and is based on a TOF count of over two out of four responses, is rudimentary [10].

Our current investigation has several limitations. The first limitation lies in the observational, single-arm design, rendering it difficult to validate our proposal of using a T1/Tc cut-off of 0.6 in patients who exhibit neurological injuries during surgery. However, this limitation is difficult to overcome because of ethical concerns. Secondly, the sample size was determined empirically because this investigation was exploratory. This approach may have lacked power calculation, potentially affecting the generalizability of the results. Thirdly, this study is a single-group study. Single-group studies are susceptible to selection bias and pose challenges in controlling confounding factors. Generalizing the results of this study may be hindered as a result.

## 5. Conclusions

The T1/Tc and MEP amplitudes showed similar sensitivity to NMB, and both decreased approximately proportionally. To ensure accurate MEP amplitudes monitoring, it is considered most ideal to conduct MEP monitoring with NMB agents fully reversed; however, if its use is unavoidable, maintaining a consistent blockade level is crucial. Based on our findings, we recommend that the T1/Tc ratio be kept greater than 0.6. This recommendation is supported by our observation that partial NMB with a T1/Tc ratio higher than 0.6 is associated with only minor increases in the CV and is therefore acceptable for maintaining reliable MEP amplitudes.

## Figures and Tables

**Table 1:** Patient characteristics.

Variables	Values
Demographic characteristics	
Sex, n (%)	
Male	41 (41.0)
Female	59 (59.0)
Height, cm, mean ± SD	163 ± 8
Weight, kg, mean ± SD	64 ± 12
Body mass index, kg/m^2^, mean ± SD	24 ± 4
Concomitant disease, n (%)	
Hypertension	31 (31.0)
Diabetes mellitus	13 (13.0)
Chronic kidney disease	4 (4.0)
Coronary artery disease	3 (3.0)
Anesthetic characteristics	
Anesthesia duration, min, mean ± SD	397 ± 186
Position, n (%)	
Supine	54 (54.0)
Lateral decubitus, right	15 (15.0)
Lateral decubitus, left	28 (28.0)
Prone	3 (3.0)
Anesthetic agents, mean ± SD	
Propofol, mg/kg/hr	6.6 ± 1.6
Remifentanil, µg/kg/hr	8.8 ± 2.3
Phenylephrine, µg/kg/hr	12.7 ± 13.9

SD, standard deviation.

**Table 2:** Parameter estimates of the PD models.

Variables	Point Estimation (%RSE)		
	Baseline, μ	EC_50_, μM	Hill Coefficient
T1/Tc			
Population mean	Not applicable	2.69 (2.62%)	2.23 (4.98%)
SD of BSV		0.153 (17.7%)	0.39 (12.2%)
Del			
Population mean	366 (10.5%)	3.05 (7.06%)	2.67 (6.31%)
SD of BSV	0.976 (7.77%)	0.59 (9.36%)	0.485 (10.5%)
SD of WSV	0.0436 (57.0%)	-	-
APB			
Population mean	1520 (9.62%)	2.64 (7.92%)	2.03 (8.03%)
SD of BSV	0.883 (7.8%)	0.647 (9.86%)	0.678 (8.95%)
SD of WSV	0.0549 (17.1%)	-	-
TA			
Population mean	738 (11.2%)	2.46 (7.62%)	2.43 (9.22%)
SD of BSV	1.07 (8.37%)	0.648 (9.62%)	0.808 (8.47%)
SD of WSV	0.0915 (13.8%)	-	-
AH			
Population mean	737 (11.7%)	1.83 (12.6%)	1.59 (7.56%)
SD of BSV	1.09 (8.79%)	1.03 (11.7%)	0.596 (11.2%)
SD of WSV	0.124 (12.4%)	-	-

PD, pharmacodynamics; %RSE, relative standard error (%); µ, mean MEP amplitude; EC_50_, half-maximal effective concentration; T1, first evoked response to train-of-four stimulation; Tc, control response before rocuronium infusion; SD, standard deviation; BSV, between-subject variability; Del, deltoid; WSV, within-subject variability; APB, abductor pollicis brevis; TA, tibialis anterior; AH, abductor hallucis.

**Table 3:** Analysis of CV associated with MEP amplitude.

Parameters	Del	APB	TA	AH
Estimation result of the bi-exponential model				
Fixed effect (%RSE)				
$\alpha_{1}$	0.211 (16.1%)	0.281 (11.2%)	0.3 (16.2%)	0.272 (9.56%)
$\alpha_{2}$	0.195 (12.1%)	0.197 (6.52%)	0.213 (10.1%)	0.275 (7.84%)
$\mu_{50,1}$	290 (33.5%)	596 (17.0%)	410 (21.7%)	122 (57.8%)
$\mu_{50,2}$	4588 (18.6%)	94,800 (48.8%)	15,200 (112%)	13,300 (55.8%)
Random effect (%RSE)				
$\alpha_{1}$	0.531 (26.1%)	0.437 (32.0%)	0.553 (22.1%)	0.269 (47.1%)
$\alpha_{2}$	0.139 (47.3%)	0.167 (30.1%)	0.159 (29.0%)	0.135 (31.0%)
$\mu_{50,1}$	0.958 (25.0%)	0.822 (19.3%)	1.02 (19.0%)	2.03 (33.5%)
$\mu_{50,2}$	0.552 (35.0%)	1.04 (80.9%)	0.862 (69.0%)	0.925 (32.7%)
Prediction of MEP amplitudes (mV) associated with target CVs				
Target CVs				
25%	264	972	757	1268
30%	139	591	478	267
35%	62	359	309	152

CV, coefficient of variance; MEP, motor-evoked potential; Del, deltoid; APB, abductor pollicis brevis; TA, tibialis anterior; AH, abductor hallucis; %RSE, relative standard error (%); $\alpha_{1}$, maximum amplitude of the first exponential, $\alpha_{2}$, maximum amplitude of the second exponential, $\mu_{50,1}$, MEP associated with 50% decay of the first exponential, $\mu_{50,2}$, MEP associated with 50% decay of the second exponential.

## Data Availability

The data of this investigation is shared in Mendeley Data [11].

## Supplementary Materials

The following supporting information can be downloaded at: https://www.mdpi.com/article/10.3390/jcm13154281/s1, Figure S1: Flow diagram; Figure S2: Monte Carlo simulations of individual MEP amplitudes (top) and population means (bottom) for the four muscles; Figure S3: Theoretical relationships between mean MEP amplitude (μ) and T1/Tc given different relative magnitudes of EC_50_ and Hill coefficients; Table S1: Population PK parameter estimates used for fitting the PKPD model of rocuronium; Table S2: Summary statistics of MEP amplitudes stratified by T1/Tc intervals.
