# Supplementary material for: Quantitative Analysis of the Effect of Neuromuscular Blockade on Motor-Evoked Potentials in Patients Undergoing Brain Tumor Removal Surgery: A Prospective, Single-Arm, Open-Label Observational Study
Source: J Clin Med. 2024 Jul 23;13(15):4281. doi: 10.3390/jcm13154281 (PMC11312558; doi:10.3390/jcm13154281)
Supplement: Supplementary file 1 [file jcm-13-04281-s001.zip › jcm-3075259-supplementary.pdf]

SUPPLEMENTARY MATERIAL

Pharmacokinetic-pharmacodynamic model of rocuronium

The model equations are as follows:

$$\frac{d(R_c)}{dt} = -\frac{CL}{V} \cdot R_c - \frac{Q}{V} \cdot R_c + \frac{Q}{V2} \cdot R_p \qquad \text{... (S1)}$$

$$\frac{d(R_p)}{dt} = \frac{Q}{V} \cdot R_c - \frac{Q}{V2} \cdot R_p \qquad \text{... (S2)}$$

$$\frac{d(C_e)}{dt} = k_{e0} \cdot (\frac{R_c}{V} - C_e), \qquad \text{... (S3)}$$

where R_c and R_p represent the amount of rocuronium in the central and peripheral compartments, respectively. C_e represents the effect-site concentration of rocuronium. CL, V, Q, and $V2$ denote the elimination clearance, central volume of distribution, distribution clearance, and peripheral volume of distribution of rocuronium, respectively. k_{e0} is the rate constant of drug transfer in and out of the effect-site compartment.

Inhibitory sigmoid E_{max} models were used to link C_e with the T1/Tc and MEP amplitudes μ.

$$T_1 = \frac{T_c}{1 + \left(\frac{C_e}{EC_{50,T_1/T_c}}\right)^{\gamma_{T_1/T_c}}} \qquad \text{... (S4)}$$

and

$$\mu = \frac{\mu_0}{1 + \left(\frac{C_e}{EC_{50,MEP}}\right)^{\gamma_{MEP}}} \qquad \text{... (S5)}$$

where $EC_{50,T_1/T_c}$ and $EC_{50,MEP}$ are the effect-site concentrations of rocuronium associated with half-maximal suppression of the T1/Tc and μ, respectively, and γ_{T_1/T_c} and γ_{MEP} are Hill coefficients associated with the corresponding endpoints. μ_0 represents the baseline (and the maximum) MEP amplitude on the unaffected side (i.e. limbs ipsilateral to brain tumor location).

Based on Equations (S4) and (S5), the functional relationship between the T1/Tc and μ can be derived as follows:

$$\mu = \frac{\mu_0}{1 + (EC_{50,r} \cdot \left(\frac{1 - T_1/T_C}{T_1/T_C}\right))^{\gamma_r})} \quad \ldots \text{(S6)}$$

where $EC_{50,r} = \frac{EC_{50,T_1/T_C}}{EC_{50,MEP}}$ and $\gamma_r = \frac{\gamma_{MEP}}{\gamma_{T_1/T_C}}$.

Non-linear regression models of coefficient of variation (CV) on mean MEP amplitude

The model equations are as follows:

Model I (mono-exponential model)

$$\frac{\sigma}{\mu} = \alpha \cdot e^{-\frac{\mu}{\mu_{50}}} \quad \ldots \text{(S7)}$$

Model II (biexponential model).

$$\frac{\sigma}{\mu} = \alpha_1 \cdot e^{-\frac{\log(2)}{\mu_{50,1}} \cdot \mu} + \alpha_2 \cdot e^{-\frac{\log(2)}{\mu_{50,2}} \cdot \mu} \quad \ldots \text{(S8)}$$

The following equation was used to calculate the MEP amplitudes corresponding to different target CVs:

$$\text{Target CV} = \alpha_1 \cdot e^{-\frac{\mu}{\mu_{50,1}}} + \alpha_2 \cdot e^{-\frac{\mu}{\mu_{50,2}}} \quad \ldots \text{(S9)}$$

Supplementary figures

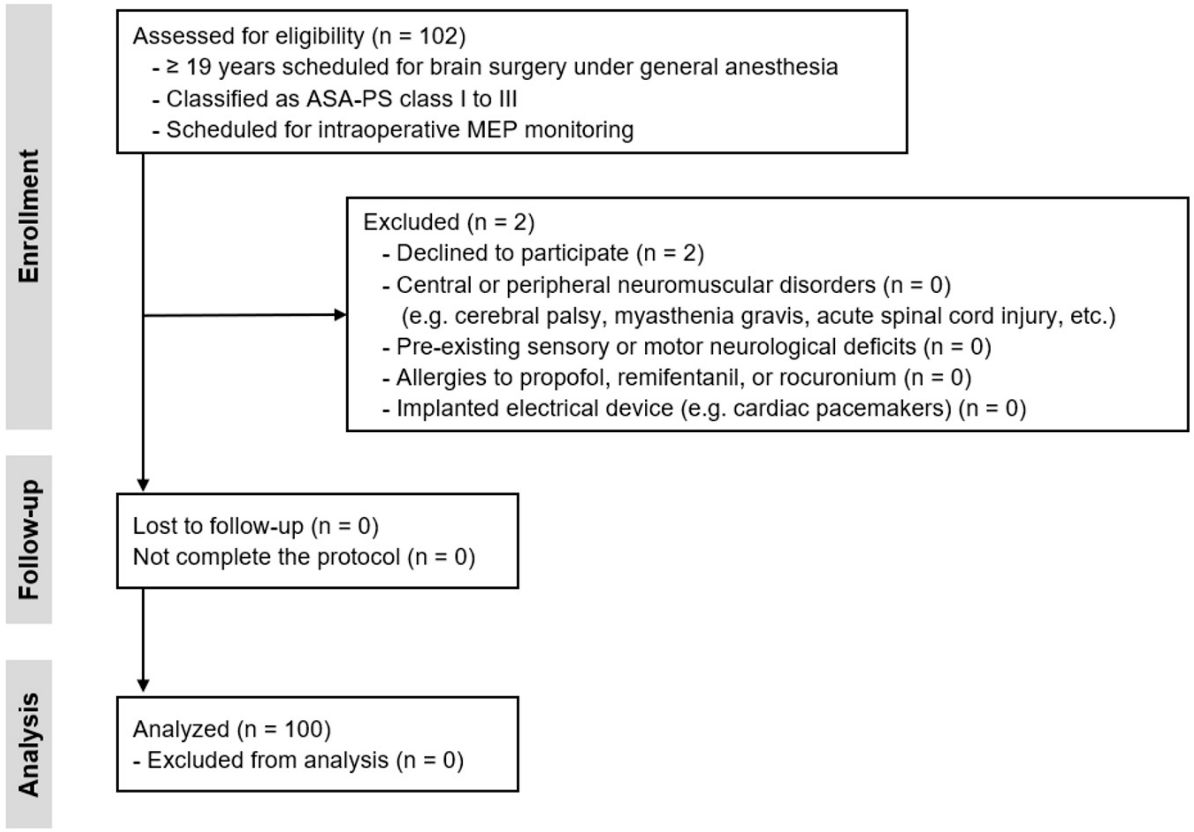

Figure S1. Flow diagram.

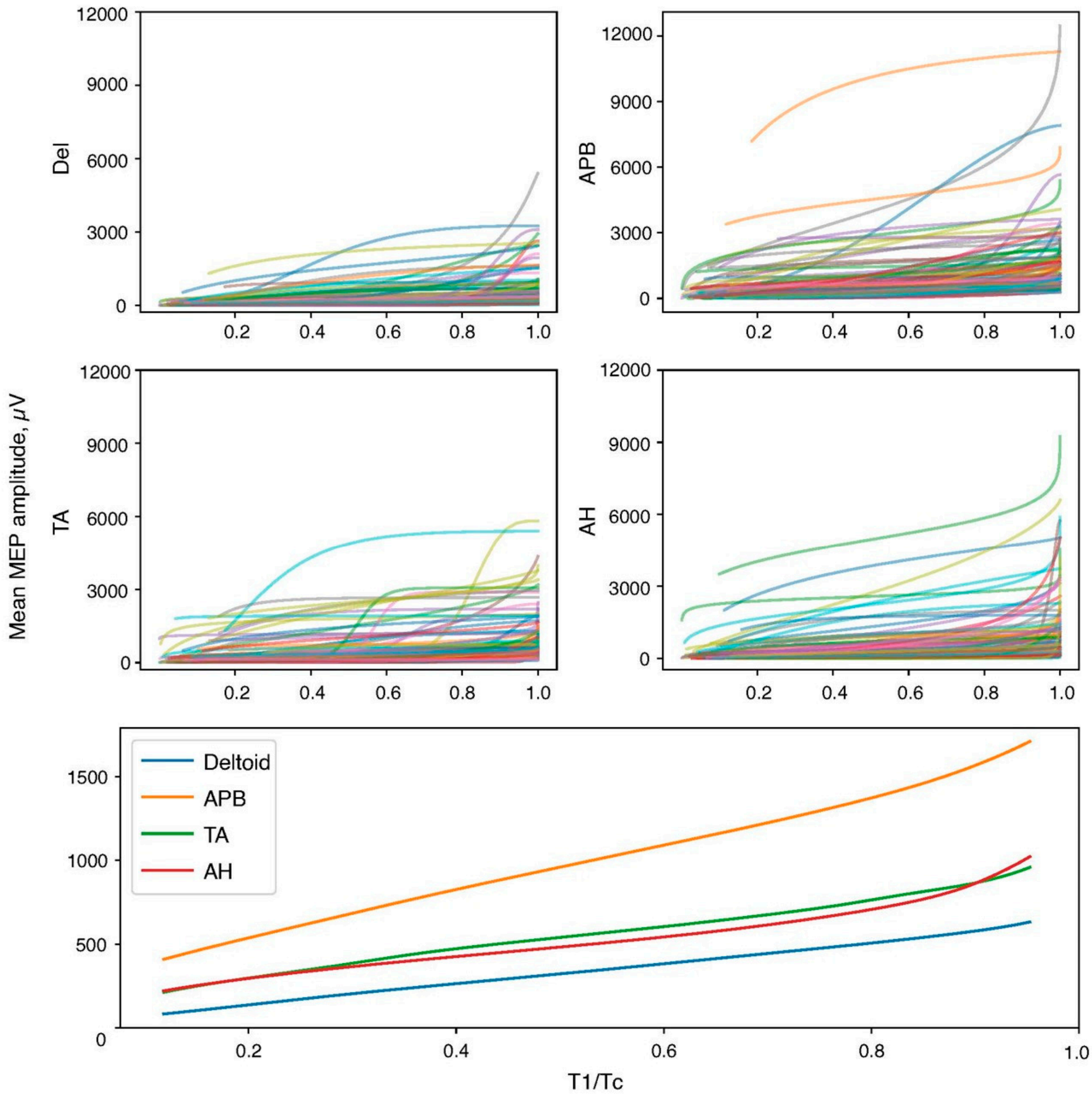

Figure S2. Monte Carlo simulations of individual MEP amplitudes (top) and population means (bottom) for the four muscles

MEP, motor-evoked potential; Del, deltoid; APB, abductor pollicis brevis; TA, tibialis anterior; AH, abductor hallucis; T1, first evoked response to train-of-four stimulation; Tc, control response before rocuronium infusion.

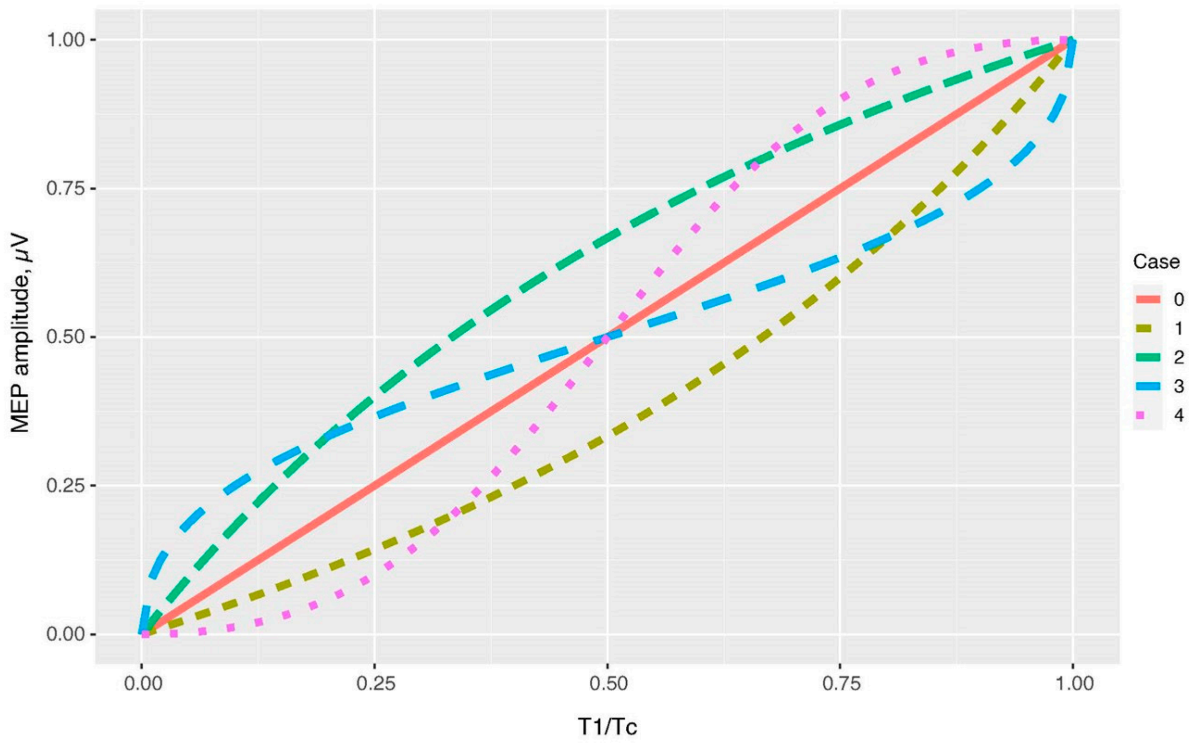

Figure S3. Theoretical relationships between mean MEP amplitude (μ) and T1/Tc given different relative magnitudes of EC_{50} and Hill coefficients

Case 0: $EC_{50,r}$=1, γ_r=1, Case 1: $EC_{50,r}$<1, γ_r=1, Case 2: $EC_{50,r}$>1, γ_r=1, Case 4: $EC_{50,r}$=1, γ_r<1, Case 5: $EC_{50,r}$=1, γ_r>1. For definitions of $EC_{50,r}$ and γ_r, see Methods.

MEP, motor-evoked potential; T1, first evoked response to train-of-four stimulation; Tc, control response before rocuronium infusion.

Supplementary tables

Table S1. Population PK parameter estimates used for fitting the PKPD model of rocuronium

Parameters	Fixed effect	Random effect
CL (L/min)	0.27	0.32
V (L)	4.73	0.24
Q (L/min)	0.22	0.32
V2 (L)	6.76	0.42
ke0 (/min)	0.13	-

PK, pharmacokinetics; PKPD, pharmacokinetics-pharmacodynamics.

Table S2. Summary statistics of MEP amplitudes stratified by T1/Tc intervals

T1/Tc	0–0.2	0.2–0.4	0.4–0.6	0.6–0.8	0.8–1.0
Del					
Mean	93	198	378	437	572
Median	55	118	218	268	409
Max	515	1360	2245	2657	2792
Min	2.2	8.8	9.2	10.8	24.8
APB					
Mean	361	686	1003	1353	1816
Median	213	508	696	1031	1422
Max	2862	3274	4295	7329	6246
Min	3.8	7.8	9.8	13.5	17.8
TA					
Mean	122	334	541	703	848
Median	71	220	313	497	466
Max	627	1613	2467	2450	3540
Min	1.4	3.8	2.7	2.2	7.4
AH					
Mean	146	261	428	513	741
Median	89	178	294	371	472
Max	999	1108	1743	2461	3670
Min	2.6	2.5	2.5	1.7	4.6

MEP, motor-evoked potentials; Del, deltoid muscle; APB, abductor pollicis brevis muscle; TA; tibialis anterior muscle; AH, abductor hallucis muscle
